# “To do or not to do”, male nurses’ experiences of providing intimate care to female patients in China, a constructivist grounded theory study

**DOI:** 10.1186/s12912-024-01896-y

**Published:** 2024-04-02

**Authors:** Xiao-Chen Lyu, Xiang-Yun Sun, Li-Hung Lee, Cheng-I Yang

**Affiliations:** 1 The Outpatient Operating Room, First Affiliated Hospital of Wannan Medical College, Anhui, China; 2 The Department of Geriatric, First Affiliated Hospital of Wannan Medical College, Anhui, China; 3 Department of Nursing, Hungkuang University, No. 1018, Sec. 6, Taiwan Boulevard, Shalu District, 433304 Taichung, Taiwan

**Keywords:** Male nurse, Experiences, Intimate care, Constructivist grounded theory

## Abstract

**Background:**

Some studies suggest that female patients have more concerns about receiving intimate care from male than female nurses. Thus, providing intimate care to female patients is a challenging experience for male nurses. The purpose of this study was to explore Chinese male nurses’ experiences and process of providing intimate clinical care to female patients.

**Methods:**

A constructivist grounded theory approach was used to develop a theoretical understanding of male nurses’ experiences. This study included participants from 3 hospitals in different locations in China. Twenty-five male nurses were recruited using purposive and theoretical sampling. Semi-structured interviews were conducted. Data analysis was completed using initial coding, focused coding, theoretical coding and memo writing to produce core concepts and categories, and theory development.

**Results:**

Chinese male nurses’ experiences of providing intimate care to female patients can be constructed as a three-stage process: (1) anticipation of the level of embarrassment, (2) deciding on the process: do it or not do it and (3) protecting both parties and dealing with embarrassment. Additionally, seven themes and associated categories were identified to represent the important factors in the process of male nurses providing intimate care to female patients in China.

**Conclusions:**

Chinese traditional culture may affect the embarrassment in Chinese male nurses providing intimate care to female patients. The embarrassing situation can be divided into three different stages, and male nurses have different main concerns in each stage. Hospital nursing administrators should consider the experiences and needs of male nurses in providing intimate care and provide them with psychological support, education and training.

## Background

Nursing has historically been defined as a caring profession, and caring is viewed as a feminine trait. Despite the historical accounts of men in nursing and the growing importance of male nurses contributing to the diversity of the nursing profession, nursing remains a female-dominated profession in the current era [1]. In most countries, however, the number of men in nursing has steadily increased over the past decades. In the USA, men represent 13.3% of the nursing workforce [2]. Men account for 9.5% of nurses in Canada, 10.7% in the UK, and 7% in Australia [1]. In Asia, the proportions tend to be lower, with 4.9% in Japan and only 2.0% in China [3]. Like male nurses in most countries around the world, those in China also tend to work in emergency or critical care units. A survey of 30 tertiary general hospitals in Hunan province showed that up to 88.3% of male nurses work in operating rooms or in emergency or critical care units, with only 11.7% working in surgery and internal medicine wards [4]. Due to the characteristics of these emergency or critical care units, they are highly professional and technical, with higher salaries and more opportunities for promotion. Scholars believe that male nurses actively utilize the choice of work unit as an effective strategy for their adaptation to the nursing profession [5]. Although nursing is a female-dominated profession, male nurses are part of the nursing profession and should provide quality nursing care to all patients [1].

Nurses provide basic nursing care that requires physical and psychological closeness to diverse patients. During the execution of some basic nursing care, patients’ body parts that are considered intimate, private and sexual are exposed and touched [6]. Intimate touch has been defined as involving inspection of, and possible physical contact with [7], a patient’s private parts. Nurses touch patients in order to perform clinical tasks. The areas of the body that are touched during intimate care provision include the genitalia, buttocks, perineum, inner thigh, lower abdomen and breasts [8]. Nurses touch areas of the patient’s body such as the breasts for electrocardiograms, the genitalia for catheterization, the buttocks for intramuscular injection, the perineum for washing, etc [7]. During intimate care, a nurse and a patient need to establish a relationship in a confined space, where a patient has to grant a stranger access his/her body’s most intimate parts and, in turn, the nurse has to see and touch another person’s body [6]. Although the physical exposure and touching of these patients are necessary steps in the therapeutic and caring process, such “task-oriented touch” can cause stress and may bring about feelings of anxiety, embarrassment and discomfort for both nurses and patients [7], especially when a nurse is caring for the opposite sex [9].

There are not many relevant studies in the literature on male nurses providing intimate care to female patients. What is evident from the literature is the challenging and problematic nature of male nurse touch, specifically the perception of sexualized touch in the provision of body care [10]. During the process of receiving intimate care, female patients might worry about male nurses looking at and touching their bodies [10]. At the same time, providing intimate care to female patients poses challenges and triggers unpleasant experiences for the male nurses themselves [8]. Male nurses worry that performing this task-orientated touch on specific areas of the female patient’s body might be misinterpreted by the patients as having a sexual purpose [9].

Additionally, the attitude of female patients toward receiving intimate care from male nurses may also be influenced by cultural factors. In the West, male nurses are offered more opportunities and autonomy to take care of female patients, whereas the duties of Chinese male nurses are greatly reduced by traditional culture norms [3]. The traditional Chinese cultural view holds that men and women are different in physiology and psychology, so the etiquette that should be observed should be different, and the communication between men and women should also be cautious [11]. The Chinese are influenced by Confucianism, and according to the Analects of Confucius, people are advised not to look at what is contrary to propriety, not to listen to what is contrary to propriety, not to speak what is contrary to propriety, and not to do what is contrary to propriety. Previous research has shown that despite society’s progress into modernity, traditional Chinese culture is still deeply rooted in college students’ attitudes toward the exposure of women’s bodies and intimate relationships. To college students educated in the West, the sexual culture in modern Chinese society would seem relatively conservative [12]. Additionally, it has been argued that male nurses in China may experience the unwillingness of female patients to receive intimate care from male nurses because of the traditional Chinese culture [13].

According to the information we have, most hospitals across the country, at least in the central provinces, recommend that male nurses be accompanied by female nursing colleagues and communicate appropriately when providing intimate care to female patients, but these are only superficial rules. There is a lack of relevant clear regulations or provision of education and training courses on intimate care to male nurses. Such advice and strategies may protect patients and male nurses from misunderstandings or conflicts during the care process and reduce possible subsequent legal disputes. But this may be of limited help in how male nurses can reduce the embarrassment of providing care to female patients. In view of this, it is necessary for us to deeply understand the experience of male nurses when providing intimate care to female patients, understand their needs, and then try to reduce their distress and improve the quality of care.

Although the literature has highlighted the importance of gender and sexuality issues related to intimate care, male nurses who are particularly disturbed by intimate care should be a focus of future research [9]. There is very limited literature on the challenges encountered by Chinese male nurses in providing intimate care to female patients. This study intends to use the method of qualitative research (constructive grounded theory) to understand the relevant experiences of Chinese male nurses in providing intimate care to female cases, especially the process of providing intimate care, the influential factors and the relevant context. The purpose of this study was to explore Chinese male nurses’ experiences and interaction processes of providing intimate care to female patients in a clinical setting.

## Methods

### Design

This study was guided by the work of [14], and a constructivist grounded theory approach was adopted. Constructivist grounded theory takes on a methodology developed by Glaser and Strauss in 1967 and is characterized particularly by its inductive approach to research, with the aim of developing a theory or explanation grounded in the data, rather than pre-existing categories and theoretical frameworks [15]. Constructivist grounded theory is based on interpretivist, subjectivist assumptions; its methodological underpinnings focus on how participants construct meaning in relation to the area of inquiry, and a constructivist co-constructs experience and meanings with participants [16]. As a research method, constructivist grounded theory is ideal when little is known about a topic because it offers explanatory power beyond description as to what is happening in practice [14]. The interpretive nature of the constructivist grounded theory approach allows for the nurse researcher to take her personal and professional experience into account, alongside the existing knowledge informing the field of enquiry [17].

### Sampling and recruitment

It was anticipated that the findings would have wider relevance if data were collected from more than one clinical setting and spanned a variety of nursing specialties [17]. Therefore, in this study, the male nurse participants were recruited from different wards or units in hospitals in different locations in China. The inclusion criteria for participants were as follows: age of 20 years or more, male nurse license in China, at least 6 months of work experience in nursing and experience in providing intimate care to female patients in the clinic. Participants were invited and clearly informed of their rights and obligations before they decided to participate in this study, and informed consent was obtained from all participating male nurses before the interview.

Purposive sampling was used to locate participants who could share their experiences and perspectives on providing intimate care to female patients because it allowed for intentional locating of participants who could provide rich data on the topic of interest [18]. Initially, a recruitment poster was sent via email to the research departments and nursing heads of hospitals A and B. Both hospitals were Grade A tertiary hospitals located in Wuhu City, Anhui Province, in eastern China. A list of 25 potential participants who met the inclusion criteria and expressed interest was returned, with the potential participants’ agreement. All were recruited, but four later withdrew due to scheduling conflicts and personal reasons, leaving 21 participants in the initial stage of the study.

Additionally, theoretical sampling, as suggested by [14], was used as the study progressed in the later stage. Additional data were sought based on concepts developed from the initial data analysis, and the participants were asked to enrich the emerging theory [17]. For example, from the analysis of the first 21 participants’ experiences, we drew up preliminary theoretical constructs. We wanted to further confirm whether the initial theoretical framework could also be applied to male nurses at hospitals in different provinces. Through introductions, we recruited 4 participants who worked at hospital C, a Grade A tertiary hospital located in Chengdu City, Sichuan Province, in western China. These participants were in the same age group as the previous 21 participants. Through interviews, we learned about their experiences with female patients, who were perceived to be influenced more by traditional Chinese culture. Although they seemed to encounter more female patients who were concerned about receiving intimate care from male nurses, the experiences of these 4 male nurses were in line with the initially developed theory. After 25 interviews, we judged that theoretical saturation had been achieved; i.e., through the data analysis, no new properties of our theoretical categories emerged. Therefore, data collection ceased.

### Data collection

Data were collected via semi-structured interviews, which took place in conference rooms in clinical settings. Each participant was interviewed once. All interviews were conducted by the first author, lasted a range of 30 min to one hour, and were audio recorded and transcribed verbatim. The questions asked during the interviews are: (1) What are your experiences and thoughts regarding providing intimate care to female patients in the clinic? (2) What you usually do when you provide nursing care to female patients and you need the patient to expose her private parts or you need to touch her body or private parts? (3) Do you feel comfortable providing intimate care to female patients? (4) Is there anything that you find difficult or disturbing? If so, how do you deal with it? The interview questions were developed based on the research topic, purpose, and personal experiences, as well as modified from a previous study that we believed would contribute to a more complete understanding of the participants’ relevant experiences [19]. Additionally, during the interview process, the interviewer tried to encourage the participants as much as possible to share their experiences so that we could understand the research phenomenon, and the interviewer did not deliberately interfere or direct the content or ways of sharing of the participants.

### Data analysis

In this study, data were analyzed using the method proposed by Charmaz [14]. This method includes the following elements: initial coding, focused coding, theoretical coding and memo writing. Initial coding is the first level of analysis and is done line by line to find potential categories and processes by opening up the raw data to as many possibilities as present themselves. In this phase, we tried our best to determine the codes for the meaningful experiences, thoughts or opinions on providing intimate care to female patients shared by male nurses. Focused coding is applied when initial codes stand out as significant or appear frequently in the initial coding, and it involves sorting, synthesizing and organizing codes and large amounts of data [20]. We examined all codes to determine their frequency of occurrence, times, and event contexts, and we aimed to clarify the relationships between them to form categories. For example, during this phase of analysis, we confirmed that three factors— environmental factors, patient factors, and body parts—were related to male nurses’ self-anticipation of acceptance by a patient/family before providing intimate care. The next step is theoretical coding, the purpose of which is to identify potential relationships between categories developed earlier, so as to work out an analytical narrative that could lead towards a theory [14]. We discussed the codes and categories, drew a figure and visualized their relationships based on the factors of time and context. In this phase, for example, we confirmed that the process of a male nurse providing intimate care to female patients can be divided into three stages, as follows: stage one, anticipation of the level of embarrassment; stage two, deciding on the process/to do it or not to do it; and stage three, protecting both parties and dealing with embarrassment. We also confirmed the categories and codes of the three stages. Additionally, Charmaz suggests including note-taking or memos as part of the cyclical process to help construct the primary categories [14]. In this study, the early stage of memo taking focused on what we saw happening in the data. Additionally, we focused on what process was at issue and under which conditions this process developed. The later stage of memo taking focused on making comparisons, such as comparing different participants’ accounts or experiences and comparing categories in the data with other categories [14].

We used the qualitative data analysis software NVivo 14 for Windows to manage and analyze the data. The analytical process was led by the fourth author. Analysis of focused codes and emerging categories were carried out jointly with all authors. The near-final version of the results was presented graphically to 3 study participants at each hospital. They appreciated the presentation of the results, stating that the findings were consistent with their experiences in providing intimate care to female patients.

### Rigor and trustworthiness

All authors, having worked as nurses for many years, had worked with male nurses and had expertise in doing qualitative research. Notably, the first and fourth author of this study are both male nurses. In their past clinical experiences, they had provided intimate care to female patients. This was helpful for us to understand the experiences shared by the participants. They also often reflected on their own experiences and the phenomena they had observed and discussed them with the other two researchers to avoid subjectivity when interpreting the experiences of the participants, which would have affected the research results. Additionally, several strategies were employed to establish rigor and ensure the trustworthiness of this research. Member checking, during which the researchers returned to participants and asked them to check and comment on the transcripts with the aim of validating the interviews, strengthened the study’s credibility [21]. Peer review among the authors was also performed to offer a mechanism for validating the research findings [20]. Additionally, we used the CASP qualitative checklist; the 10 questions and their suggested belonging considerations in the checklist were used to examine the whole manuscript to enhance its trustworthiness and the quality [22].

## Results

The participants in this study included 25 male nurses from three hospitals located in two different provinces of China. Their age ranged from 22 to 37 years (mean = 28.5). Most were aged less than 30 years (18; 72%) and had bachelor degrees in nursing (21; 84%). The participants came from many different nursing workplaces. Most of them (10; 40%) served in general medical or surgical wards, followed by intensive care units (7; 28%). The others were from the emergency room, operating room, and obstetrics and gynecology wards. Their job experience in nursing ranged from 0.5 to 14 years (mean = 5.4). Table 1 shows the detailed demographic characteristics of the participants. Additionally, besides the basic nursing skills learned at university or college, none of the participants had received relevant education or training on how to provide intimate care to female patients after graduation.

**Table 1:** Participant characteristics (*N* = 25)

	Numbers	Percentages
Age (year)	Range = 22–37	Mean = 28.5
20–25 26–30 31–35 36–40	9 9 4 3	36% 36% 16% 12%
Degree		
Associate Bachelor Master Doctor	2 21 1 1	8% 84% 4% 4%
Ward /Unit		
General medical or surgical ICU ER OR Obstetrics and Gynecology	10 7 3 2 2	40% 28% 12% 8% 8%
Hospital		
A in Anhui Province B in Anhui Province C in Sichuan Province	16 5 4	64% 20% 16%
Nursing work experience (Year)	Range = 0.5–14	Mean = 5.4
≥ 10 years 6–10 years 3–5 years 1–2 years ≤ 1 year	4 7 7 1 6	16% 28% 28% 4% 24%

According to the participants’ experiences, we found that these male nurses basically believed that medical care should not be gender-specific. They felt that, as nurses, they should provide any care for female patients, including intimate care. However, considering that patients and family members may be influenced by traditional Chinese culture, they may have concerns and scruples about receiving care from male nurses. Therefore, decisions on implementing intimate care for female patients should take into account not only the standard nursing skills but also the patient’s feelings, and nurses should respect the wishes of patients and family members. The participants made efforts to reduce the patients’ and their own embarrassment while protecting both parties to avoid misunderstandings and disputes during the process of implementing intimate care for female patients.

**Fig. 1:**
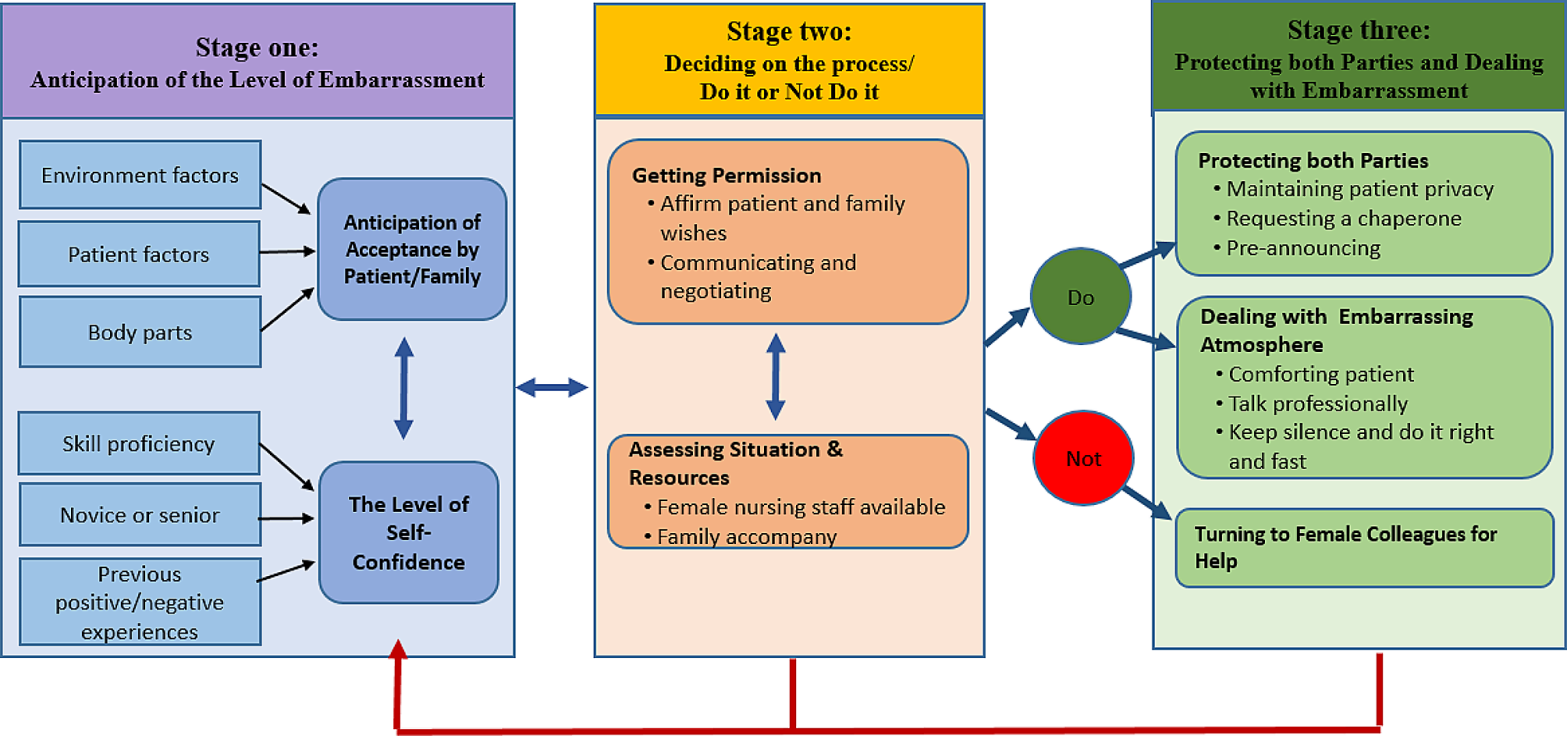
Framework on the process of male nurse providing intimate care for female patients

From the data analysis, three stages, seven themes and associated categories were identified to represent the process of male nurse providing intimate care to female patients in China, as follows: **Stage one**: anticipation of the level of embarrassment, and its belonging themes of “anticipation of acceptance by patient/family” and “the level of self-confidence”. **Stage two**: deciding on the process/do it or not do it and the two themes of “getting permission” and “assessing situation and resources”. **Stage three**: protecting both parties and dealing with embarrassment and its three themes of “protecting both parties”, “dealing with the embarrassing atmosphere” and “turning to female colleagues for help”. Additionally, each of the 7 themes also contained 2–3 categories, for a total of 16 categories. The details of the 3 stages, 7 themes and 16 categories and their relationships are provided in Table 2; Fig. 1. Each theme is illustrated by representative quotes from participants in the following sections.

**Table 2:** Stages, themes and categories

Stage	Themes	Categories
Stage one: Anticipation of the level of embarrassment	Theme 1: Anticipation of acceptance by patient/family	• *Environmental factors* • *Patient factors* • *Body parts*
	Theme 2: The level of self-confidence	• *Skill proficiency* • *Novice or senior* • *Previous positive/negative experiences*
Stage two: Deciding on the process/Do it or not do it	Theme 3: Getting permission	• *Affirming patient and family wishes* • *Communicating and negotiating*
	Theme 4: Assessing situation and resources	• *Female nursing staff available* • *Family member accompany*
Stage three: Protecting both parties and dealing with embarrassment	Theme 5: Protecting both parties	• *Maintaining patient privacy* • *Requesting a chaperone* • *Pre-announcing*
	Theme 6: Dealing with embarrassing atmosphere	• *Comforting the patient* • *Talking professionally* • *Remaining silent and doing it right and fast*
	Theme 7: Turning to female colleagues for help	

### Stage one: anticipation of the level of embarrassment

When the male nurses know they have to perform an intervention for a specific female patient that involves the female patient’s private parts, in the first stage, they tend to anticipate the embarrassing situation and degree of embarrassment they may face when conducting the intimate care. The sense of embarrassment is influenced by male nurses’ prediction on the acceptance of the patient or family members toward their care and also their self-confidence in conducting intimate care.

### Theme 1: anticipation of acceptance by patient/family

For the male nurses in this study, whether patients and family members could accept them performing this intimate care was the main source of embarrassment. According to their experience, environmental factors (hospital location; the ward or unit), patient factors (age, conscious state, severity of illness, marital status, education level, etc.), and the body parts to be exposed all impact the acceptance of patients and their family.

#### Environment factors

The province, city or county where the hospital is located may, though not always, affect the education level of female patients and the influence of traditional Chinese culture on patients and their families. In addition, the ward unit where the male nurse serves is also an influencing factor. If it is the emergency room, intensive care unit, or operating room, the patient’s condition is usually more serious. At such times, patients and family members tend to be more accepting of necessary treatment, even if it involves exposure of private parts. In contrast, if the setting is a general medical and surgical ward, patients and family members have more time for consideration, and therefore male nurses may face more challenges.

*Our hospital is located in a relatively rural city, and the education level of the patients may be relatively low. Some female patients may have more concerns about accepting care from male nurses. This is also a challenge for us male nurses. (Participant 1)*

*Basically, the patients in our intensive care unit, they know that they are here, and their condition is relatively serious, so basically, they are more able to accept our treatment and care. (Participant 7)*

#### Patient factor

The female patient’s or family’s acceptance of intimate care performed by a male nurse will vary depending on the condition of the patient. Generally speaking, if the patient is older, critically ill, or comatose or sedated, the acceptance by the patient and family members is usually higher, and the expected embarrassment perceived by male nurses is lower.

*If it is a coma patient, the treatment time should be shortened first, and rescue is the first priority…. When the patient is unconscious, the family members generally accept it. The female patient will not feel embarrassed, and neither will I. (Participant 2)*

For male nurses, the most challenging cases would be young and conscious female patients. Perhaps influenced by traditional Chinese culture, young female patients tend to feel hesitant, anxious and unwilling to expose their private body parts to a male nurse, who to them is a young male stranger.

*That young patient thinks that she is a female patient, and these treatments involving exposed private parts should be done by a female doctor or nurse. (Participant 15)*

Additionally, it is not only young female patients who have concerns about receiving intimate care from male nurses. Some male nurses (especially the younger ones) may sometimes have conflicts, psychological barriers and embarrassment when they need to provide intimate care to young female patients, as such behavior violates the traditional Chinese cultural concepts they received.

*I think it’s okay for me to care for older women, but if I meet some young women in their 20s and 30s, I think I will mind it a little bit. Because I think there is still a difference between men and women, right? Although as a medical professional, I don’t think there should be any gender distinction, but when facing female patients in their 20s and 30s, I think I still care a little psychologically. (Participant 4)*

*It is because of the similar age, I am at the same age as her. For example, if a patient is in her 20–30 s, I will feel a little strange… Yes, I will feel awkward when I see her private parts. (Participant 14)*

#### Body parts

The body parts that need to be exposed for treatment can sometimes affect the acceptance of patients. According to the participants’ experiences, patients and family members have a relatively high acceptance of the ECG with the chest exposed. However, if the patient needs to expose the perineum for catheterization or perineal irrigation, the acceptance of the patient and family members is relatively low.

*The ECG monitoring is okay; there is nothing special about the ECG monitoring. Basically, the upper body is okay for the patients, [but] if the lower body needs to be exposed, it will cause more embarrassment than the upper body. (Participant 12)*

### Theme 2: the level of self-confidence

Male nurses’ perception of embarrassment is also related to their degree of confidence in providing intimate care to female patients. According to their experiences, the self-confidence is affected by their proficiency in the skill, their work experience (novice or senior) and also their previous interactions with female patients and family when conducting intimate care.

#### Skill proficiency

Some male nurses do not have a good understanding of female anatomy and lack opportunities to practice techniques related to intimate care, which affects their self confidence in performing such tasks. For example, although participant 9 had been working as a nurse for 3 years, the nature of his work unit (trauma and orthopedic ward) meant that he had rarely needed to provide intimate care to female patients in their unit, so he had rarely had opportunities to practice. Therefore, when he occasionally encountered such a situation, he lacked confidence in whether he could perform well.

*The difficult part is that I rarely have the opportunity to perform catheterization on female patients; that is, I may have a little lack of proficiency, and I may feel that I am not familiar with the implementation. (Participant 9)*

#### Novice or senior

At the initial stage of nursing work, male nurses have relatively little experience in performing such tasks and have low self-confidence in providing intimate care to female patients. With more and more work experience, they will develop more self-confidence and experience less psychological embarrassment when facing the task of intimate care. Participant 5 (8 years of work experience) and participant 21 (12 years of work experience) compared their confidence levels in providing intimate care to female patients when they first started to engage in nursing work and now.

*At that time, I was still inexperienced, that is to say, it was quite embarrassing… maybe, it was because I had no experience in some nursing skills and related communication. (Participant 5)*

*When I started working, regarding women’s private parts, such as women’s breasts, or private parts of women… I have a little bit of scruples about different genders in my heart, but as I work for a long time, as long as the family members are willing let me to do it [then] I will do it, just treat it as a task. (Participant 21)*

#### Previous positive/negative experiences

For some male nurses, their experience and feelings of providing intimate care to female patients is a continuum. Their past obstetric practice experience at the student stage and the situations they face in clinical work after graduation both affect their confidence in providing intimate care.

*At that time, I scrubbed her body, comforted her not to be nervous, and then explained the necessity of such an operation. The female patient was accepting during the process. At that time, I felt that I had learned a new nursing skill, and I was very happy. If I encounter this kind of patient again, I may not worry anymore. (Participant 12)*

*She said that she didn’t want a boy to do the electrocardiogram for her. In the end, I asked a female nurse to do it. I felt a little bit rejected. This experience probably affected my mood at work for a period of time. I had that thought of never wanting to provide this kind of care to a female patient ever again. (Participant 14)*

#### Stage two: deciding on the process/Do it or not do it

After feeling the anticipated degree of embarrassment, the male nurse will enter the second stage, the process of deciding whether to implement the intimate care himself. At this stage, the male nurse will mainly respect the wishes of the female patient and family members. At the same time, the male nurse will also assess the objective conditions and the possibility of looking for other alternatives.

### Theme 3: getting permission

The wishes of patients and family members are the most important basis for male nurses to decide whether to perform a task by themselves. The process includes confirming the wishes of the patient or family members. Sometimes the patient or family members may hesitate, worried about receiving intimate care from a male nurse. They may sometimes ask for the care of female nurses. In such cases, it is necessary for the male nurse to communicate and negotiate with the patient and family members.

#### Affirm patient and family wishes

When confirming the wishes of the patients or family members, the male nurse participants first explained to the female patients or family members the examination that would be done, as well as the body parts that might involve privacy issues and exposure during the process. Then they tend to observe the facial expressions or reactions of the female patients and family members, or directly ask the patients or family members for their thoughts.

*You have to pay attention to her expression, if you feel that her expression is not very happy, we may pay a little attention. Then for this kind of patient, try to let a female nurse do it for her. (Participant 1)*

*If there is really no female nurse, I will ask her directly, I will ask her, would you like me to do an electrocardiogram for you? And if she agrees, then I’ll do it for her. (Participant 5)*

Sometimes, the wishes of female patients present an ambiguous situation, which increases the difficulty of judgment and further work arrangement of male nurses.

*I actually like it better. If the patient really wants to refuse me, I hope she can tell me directly from the beginning, because in this way I can quickly find other alternative methods.… If she says she agrees, but in fact she really minds, and then feels very embarrassed and is uncooperative during the treatment, it will cause me some trouble in my work. (Participant 10)*

#### Communicating and negotiating

Sometimes, when a patient or family member hesitates, or asks if there are other female nurses, the male nurse may spend a little more time explaining, communicating, and even coordinating with them.

*If the communication is ineffective, I will definitely call a female colleague to help, but if I am the only one on the night shift, and if I encounter a female emergency patient, I will try my best to communicate with her, saying that the treatment is urgent, be patient and explain it to her until she agrees. (Participant 4)*

Most of the participants emphasized the importance of communication skills, which not only help to reduce the anxiety of female patients and their families about receiving intimate care from male nurses but also increase the consent of patients and their families.

*If you communicate with her well, I think most of the problems can be solved during the communication process. If she minds, she will tell you during the communication. When it comes to intimate care, it will be very smooth. (Participant 9)*

### Theme 4: assess Situation & resources

The participants may also assess the situations, including whether there are a female nurse who are available and willing to provide assistance and whether the patient is accompanied by family members. This information can be referenced to decide whether to help female patients with intimate care.

#### Female nursing staff available

Female nurses are the main sources of help when male nurses encounter problems in providing intimate care. If a female nurse is free and willing to help, some of the participants are more inclined to directly seek help from the female nurse in order to avoid embarrassing situations they may face. Male nurses are especially likely to do so when they encounter young female patients and feel a high degree of embarrassment.

*For younger female patients who have never been in a relationship or married, for example, before the age of 18, or in their twenties. We usually let a female nurse care for them. We male nurses, basically, there is no need to deal with this type of patients. (Participant 14)*

*When encountering a patient in her twenties and thirties, such as doing electrocardiograms or perineal scrubbing, we usually let the female nurses do it. If there are no female nurses, only me, or when all the nurses there are male, I then do it for the patient. (Participant 5)*

Some male nurses would seek help from their female counterparts when their communication with the patient or family members was ineffective or they were rejected.

*For example, for a female patient, her boyfriend did not agree to me providing intimate care to the patient. I had already communicated with him and told him that I was a nurse, but he disagreed and there was nothing I could do, so I had to ask a female colleague to help me. (Participant 19)*

#### Family member accompany

Whether the patient is accompanied by family members is also one of the factors that male nurses consider in decisions on providing intimate care to female patients. Male nurses tend to hope that the presence of family members will be helpful for them to carry out intimate care; otherwise, they would prefer that family members do not appear.

*If there are family members at the time, wouldn’t it be better to ask the family members to accompany the patient? Especially for a couple, the husband can accompany the patient during the treatment to eliminate the patient’s doubts. (Participant 2)*

*After all, we are in the intensive care unit, and patients are usually unconscious. The presence of family members is not helpful for us to perform intimate care. Therefore, I will avoid doing some intimate care for patients when their family members are there. (Participant 7)*

#### Stage three: protecting both parties and dealing with embarrassment

Whether the reason is that the patient or family does not accept the care of a male nurse or no female nurses are available at the time, when male nurses have to perform intimate care of female patients by themselves, they will use some strategies to protect female patients and themselves and to reduce the embarrassment of both parties.

### Theme 5: protecting both parties

Protecting patients is the primary task of nursing staff. However, since intimate care involves exposure and touching the private parts of the female patient’s body, the male nurse may worry that the female patient could misunderstand the male nurse’s actions or intentions during the process, causing unnecessary trouble. Therefore, when providing intimate care, the male nurse must not only protect the patient but also do something to protect himself.

#### Maintaining patient privacy

Regardless of whether the patient is awake or not, male nurses are taught that it is important to protect the privacy of the patient when providing intimate care. Their strategies include asking family members or other people who should not be present to leave, drawing the bed curtains, and trying to minimize the exposure of the patient’s private parts during the process.

*I will pull the curtain of the patient’s bed, and then, I don’t let her breasts be fully exposed, and I try to cover some of the patient’s private parts as much as possible without affecting my work. (Participant 18)*

#### Requesting a chaperone

All male nurses in this study are required by their hospitals to find a chaperone to accompany them when they provide intimate care to female patients. This chaperone is usually a female nurse, a female family member or the husband of the female patient. It can reassure patients and protect the male nurses themselves.

*I think it is still necessary to have a female colleague with me, so that it feels more secure. For a young girl, if a female nurse is by her side, maybe the female patient’s sense of security will increase a little, and my sense of security will also increase a little. (Participant 5)*

#### Pre-announcing

For the participants, foretelling or explaining the body parts that may be exposed or touched in the next action can prevent the patient or family members from being surprised by the action performed by the male nurse, or even misunderstood as bad-intentioned.

*This is actually a way to protect the patient, and also a way to protect myself. For me, I know the procedures needed to perform intimate care, which actions may need me to touch which parts of her body, but the patient, she may not be so clear. So in this case, if I didn’t inform her in advance, it is possible that the patient may have some misunderstandings when I touch her, or even more seriously, cause some legal disputes. (Participant 10)*

### Theme 6: dealing with the embarrassing atmosphere

For participants, regardless of whether the source of embarrassment comes from the female patient or the male nurse himself, the feeling of embarrassment could affect both parties. Therefore, in the process of providing intimate care, the male nurse may use some methods to reduce the embarrassing atmosphere, such as comforting the patient, talking professionally, remaining silent, or doing it right and fast.

#### Comforting the patient

Comforting the patient refers to caring for the patient with language, telling the patient not to be too nervous, and relieving the patient’s nervousness. In this study, it usually occurs when the patient or family members have a higher acceptance of male nurses providing intimate care, and when the male nurses feel less stress.

*If the patient is awake, she may feel a little awkward. Then we would talk to her and distract her a bit to ease the awkwardness. For example, I will talk to her about her family, work, and some small things in daily life. The patient is usually more relaxed and more cooperative during the process. This is how I have been working for many years. (Participant 15)*

#### Talking professionally

Some participants may also play down the impact of gender factors on intimate care and reduce the degree of embarrassment felt by both parties through the display of professional knowledge. They talk to patients, focusing on professional topics. For example, they try to understand the reasons why patients seek medical help, explain the patient’s health conditions and the reasons for these examinations, and also provide patients with health education and so on.

*Generally speaking, I will give her a health education on some relevant knowledge of this treatment or examination, as well as the follow-up matters that patients should pay attention to, so that she can understand that I am on her side, and it is all for her good and health, and let her try to eliminate any concerns about sex. (Participant 7)*

#### Remaining silent and doing it right and fast

When the male nurses feel great stress from conducting intimate care, such as when they encounter a young female patient, it is not easy for them. Many participants tend to choose not to talk with the female patient and try to perform the action “right and fast” to end the moment of embarrassment as soon as possible and also avoid misunderstandings that might occur.

*If I know that the care I am going to do is about the private parts of the young female patient, I will be very shy, I will be very embarrassed. When I start, I will only think about how to do it. I will forget to talk to her, to comfort her. (Participant 20)*

*I feel that if I talk too much, it may cause negative feelings in some female patients. So I usually just get it done quickly and the embarrassment can end. (Participant 16)*

### Theme 7: turning to female colleagues for help

When the male nurses in this study decided to seek help from female nurses, it was usually in several situations. Sometimes they did so to respect the wishes of the patient or family members, or the male nurses might directly choose not to perform the intimate care by themselves and ask other female colleagues for help so as to avoid embarrassment. They may perform other tasks in exchange for the help from the female nurse. Some male nurses may feel regret about asking female colleagues for help.

*For some private bathing or urethral care, we ask the female colleague next door to do it, and we do other things like intravenous infusion and other routine care. (Participant 14)*

*Sometimes it is unfair to a female colleague, after all, she may be taking a break, so I would feel bad for asking her to help with this. (Participant 16)*

## Discussion

Our study explores the male nurses’ experiences and interaction process of providing intimate care to female patients in the clinic within the Chinese context and culture. Using constructive grounded theory, we interviewed 25 male nurses in different hospitals and workplaces, and from their experiences we constructed our understanding of male nurses providing intimate care to female patients. The core finding is that Chinese male nurses’ experiences of providing intimate care to female patients can be constructed as a three-stage process, as follows: (1) anticipation of the level of embarrassment, (2) deciding the process/do it or not do it and (3) protecting both parties and dealing with embarrassment. Additionally, seven themes and associated categories were identified to represent the important factors in the process of male nurses providing intimate care to female patients in China. Some critical issues around our three stages and seven themes are worthy of further discussion.

Consistent with a previous study [23], the male nurses in this study generally believe that healthcare should not be gendered and viewed providing intimate care as a fundamental and vital part of their professional responsibility as a nurse. Although it varies from person to person, not only female patients but also some male nurses may also be influenced by traditional Chinese culture. The male nurse may sometime struggle between the duties of nurses, his gender as a man and traditional Chinese culture. Therefore, when the Chinese male nurses are faced with providing intimate care to female patients, they may be hesitant to do so. The reasons they gave were mainly to respect the ideas of female patients and their family members, but there were also subtle indications that they were influenced by traditional Chinese culture. Therefore, in the face of different degrees of embarrassment in different situations, whether to do it and how to do it appropriately are important issues in intimate care.

In the first stage, before providing intimate care, the degree of embarrassment generated by the male nurse is affected by two factors, one of which is the degree of acceptance of intimate care from a male nurse that he expects of female patients and their families. In addition to the patient’s age, the patient’s health status and level of consciousness were mentioned in a previous study [24]. Among them, the age of the patient is undoubtedly the most interesting. Previous studies in many different countries, including Indonesia and South Africa, have found that male nurses feel particularly challenged and vulnerable to embarrassment when they have to provide care to young female patients [8; 24], suggesting that this is not a unique phenomenon in China but tends to be a cross-cultural phenomenon of male nurses in Asia and even in African countries. For example, in the South African context, care of the body is confined to family based on gender and occurs within the safe environment of the home [8]. But when facing older female patients, male nurses feel less pressure and less embarrassment, as they more likely to depersonalize or desexualize the situation, ignoring the gender of the patient and providing professional nursing care [23]. Our findings show that younger male nurses may sometimes have conflicts, psychological barriers and embarrassment, especially when they need to provide intimate care to young female patients. The senior male nurses in this study did not mention relevant experiences in the interviews. We think the reason may have been that the senior male nurses had more relevant experiences and knew how to deal with this type of care.

This study also found that the location of the hospital, as well as the exposed and touched body parts, may also affect the patient’s acceptance and thus indirectly affect male nurses’ embarrassment levels. We suspect that the location of the hospital may affect the education level of female patients and their family members and the degree to which they are influenced by traditional Chinese culture. Compared with their peers in acute and intensive care units, male nurses in the general ward encountered more embarrassing situations and the frequency of providing intimate care was higher. As in a previous study, most participants in this study focused on the body parts and anticipated that female patients and their family might express that such touch was unacceptable, especially on areas considered private and sensitive [8]. In addition, breast exposure was perceived as less threatening by female patients than was exposure of the perineum [25]. We suggest that these relationships should be confirmed by further research.

Male nurses’ personal self-confidence in providing intimate care also influences the degree of embarrassment they perceive. Their lack of technical proficiency in intimate care and lack of related experience are the reasons for their low self-confidence. It is not difficult to understand, as they may not be educationally prepared to interact with patients while providing intimate care to female patients [10]. The lack of implementation experience in clinical work, or negative experiences such as being rejected by patients, aggravated their lack of confidence in providing intimate care to female patients. It is also recommended that all male nurses be provided with adequate simulated pre-clinical training in providing and dealing with the process of providing intimate care [23].

In stage two, deciding on the process/do it or not do it, it is important to determine whether male nurses will personally provide intimate care to female patients. Although all the male nurses said it depended on the patient’s acceptance and they respected the decisions of the patient or family, in practice, a different attitude was shown. Some male nurses would directly choose to ask female colleagues for help to avoid embarrassment, especially when they can easily find a female colleague to help. They were only charged with providing intimate care to female patients when a chaperone and female nurse was unavailable [26]. In contrast, some male nurses were still willing to spend a little more time convincing patients and family members when they encountered hesitation from patients or family members. However, all the participants emphasized the importance of communication skills, and respect for patients requires communicating well, soliciting patient input in decisions, and honoring patient values [7]. Consistent with a previous study conducted in China, the male nurse always made the concerns of female patients their first priority, even though they were excluded from providing some kinds of intimate care to female patients [13]. In addition, most of the male nurses understood and expressed the view that the unwillingness of female patients to receive care from them could be ascribed to the traditional Chinese culture [13].

Stage three is protecting both parties and dealing with embarrassment. Consistent with previous findings [24], male nurses in this study were often apprehensive about using physical touch, and they used coping strategies to protect both parties and deal with the fear of being accused of inappropriate use of touch. When providing intimate care, male nurses were required to follow standard operating procedures, including protecting patient privacy, having a chaperone and pre-announcing, as reported in previous studies [23]. Their strategies for protecting patient privacy included pulling the bed curtains around and modifying procedural techniques to minimize patient exposure and the need for intimate touching [25; 27]. For the participants, a chaperone could be a female colleague or a member of the family. The presence of a chaperone could also provide protection against unfounded allegations of improper behavior [27]. Additionally, the need to touch female patients poses a risk for male nurses, as there is a potential for the misinterpretation of male nursing practice as sexually motivated [10]. The sexualization of male nurses’ touch provides insight into how gender stereotypes create discomfort and suspicion on the part of patients. This in turn impacts male nurses’ perceptions of their own safety while performing intimate and caregiving tasks [28]. In this study, pre-announcing was used by the male nurses as a strategy to reduce the possibility of being misunderstood by female patients.

Another important finding in the third stage was the strategies used by male nurses to reduce embarrassment in both patients and themselves during intimate nursing care. It has been argued that during the process of intimate care, both female patients and male nurses experience a feeling of discomfort, and that if a patient feels uncomfortable, then the male nurses should feel uncomfortable as well [25]. In the way of reducing embarrassment, what is more interesting is whether the nurse should talk to the patient. Our research results show that a male nurse should say something to comfort a female patient, whether he is lightly chatting with the patient or talking about health knowledge in a professional manner, to divert the female patient’s attention. Such diversion can make the patient feel less stress and more confident about the situation. Some male nurses are under high pressure. Especially when young male nurses meet young female patients, they may be very nervous, unconfident, or worried that their speech will be misunderstood by the female patients, so they choose not to speak during the process, as they just want to hurry and get things done. This method may help them quickly escape the embarrassing situation, but it may ignore the feelings and needs of female patients [29].

When the male nurse wanted to avoid embarrassment or was rejected by the female patient, asking a female nurse to help perform intimate care was the main alternative. However, there are still some hidden concerns, such as the general shortage of clinical nursing manpower or the high proportion of men in the workplace, which will inevitably increase the difficulty of finding a female nurse to perform intimate care. Therefore, hospital administrators must understand the challenges and needs of male nurses when providing intimate care to female patients and assist them with necessary education, training, and psychological support [24]. For example, raising awareness of intimate care conflicts will assist them in developing strategies to deal with uncomfortable intimate care incidents without losing their identity as men. They should be encouraged to reflect on their sociocultural values and be open to listening and learning from their patients [8].

### Nursing implications

In view of the above, the findings of this study shed light on the situations and problems that male nurses may face when caring for female patients and providing intimate care. The experiences shared by senior male nurses and the effective strategies they adopted can also provide a reference for new male nursing staff or male nursing students when they encounter similar situations in clinical practice. In addition, the findings of this study will help nursing managers and nursing educators to guide male nurses and provide necessary psychological counseling and educational training, and they provide a reference for developing relevant policies.

### Limitation

As with all research, the limitations of this study must be acknowledged. First, this study was a constructivist grounded theory study informed by symbolic interactionism; accordingly, it assumes that reality is socially constructed through actions and interactions of individuals and society. Subsequently, the findings reported are only one interpretation of reality and not representative of every reality. In the participant recruitment process, the diversity of the sample in terms of age, nursing experience, workplace, and hospital locations was ensured as much as possible. We also achieved theoretical saturation. However, due to time and financial constraints, this study only conducted in-depth interviews with 25 male nurses, mainly in Anhui Province and a few in Sichuan Province. The absence of data from others may be construed as a limitation. Additionally, the participants might have forgotten details of events that occurred some time ago. Rather, the researchers interviewed the participants with some scripted questions and prompts so as to facilitate the participants to recall their experiences. Furthermore, future researchers should do more than just interview male nurses. It would be better to observe actual instances of intimate care or conduct interviews with other informants, such as female patients, who might provide additional perspectives that would inform the development of theory. In addition, in the future, it may be possible to design a questionnaire on the intimate care provided by male nurses based on the results of this study, and to test its reliability and validity for use as an effective research tool to conduct large-scale research on male nurses so as to detect and efficiently understand the severity of related problems.

## Conclusion

This study used constructive grounded theory to show that Chinese male nurses may be influenced by traditional Chinese culture and experience embarrassment when they provide intimate care to female patients. To avoid embarrassment, they go through a do/not-do decision process. In addition, this study also found that at different stages, male nurses have different concerns. For example, in the first stage, it is young female patients with clear consciousness who make them feel the most anxious. They may also lack relevant skills and experience, resulting in a lack of self-confidence. Then, in the second stage, they will evaluate the willingness of the patient and family members, as well as consider whether there are female nurses available to help, and decide whether to perform the intimate care themselves. In addition, communication skills are very important in the process of communicating with patients and family members. The third stage involves strategies to protect both the patient and the nurse and to minimize the embarrassment of both during the intimate care. Furthermore, the relevant hospital nursing administrators should be conscious of the experiences and needs of male nurses in providing intimate care and further provide them with psychological support in a timely manner. In addition, relevant education and training courses should be designed and arranged to provide skills related to intimate care, communication skills, methods to protect patients and themselves, and skills to avoid embarrassment during the provision of intimate care.

## Data Availability

The data that support the findings of this study are available from the corresponding author upon reasonable request.
